# A 76-year-old male with abdominal cutaneous nerve entrapment syndrome: a case report

**DOI:** 10.3389/fpain.2025.1588410

**Published:** 2025-10-31

**Authors:** Karolina Kalanj, Matija Herceg, Antonio Ivanac, Sara Kalanj, Mirta Peček, Ana Brundula

**Affiliations:** ^1^ Andrija Stampar School of Public Health, School of Medicine, University of Zagreb, Zagreb, Croatia; ^2^ School of Medicine, University of Zagreb, Zagreb, Croatia; ^3^ Institute for Emergency Medicine Virovitica—Podravina County, Virovitica, Croatia; ^4^ Polyclinic Amruševa, Zagreb, Zagreb, Croatia

**Keywords:** abdominal cutaneous nerve entrapment syndrome, abdominal pain, Carnett’s sign, nerve block, vitamin B

## Abstract

Abdominal pain represents a frequent presenting symptom in emergency departments, with up to 20% of patient visits involving abdominal and/or flank pain. Recently published studies indicate that anterior cutaneous nerve entrapment syndrome (ACNES) is a more common cause of these symptoms than previously believed, with up to 2% of patients presenting to the emergency department at teaching hospitals being ultimately diagnosed with the condition. Importantly, ACNES is often misdiagnosed as another cause of abdominal pain. We present a 76-year-old patient whose a chief complaint was persistent abdominal pain localized to the right side of the umbilicus over a 6-week period, associated with a burning sensation of the skin. Following unremarkable laboratory, imaging, and endoscopy findings, the diagnosis of ACNES was confirmed with simple Carnett's sign. This is the first documented report to highlight that interventional treatment when combined with exogenous factors can contribute to the repair of nerve damage.

## Introduction

Abdominal pain is one of the most common symptoms observed in patients arriving at emergency departments, with reports of acute abdominal pain being the primary complaint in up to 10%–20% of emergency department visits in Western Europe (1). It has been reported that at least 2% of such presented cases of abdominal pain are caused by anterior cutaneous nerve entrapment syndrome (ACNES) (2). The condition occurs when sensory nerves within the abdominal wall become pinched or entrapped within the abdominal wall muscle. In such a situation, the increase in pressure in the abdomen caused by many different reasons leads to localized, dull pain and skin burning sensation. Although the clinical diagnosis of ACNES can be straightforward, the condition is often misdiagnosed, primarily due to its rarity and limited awareness among clinicians (3). The syndrome is often misdiagnosed with intra-abdominal issues, such as irritable bowel syndrome (IBS) or somatization disorder (4, 5). This is particularly concerning, as misdiagnosis often leads to unwarranted diagnostic investigations and invasive interventions, including laparoscopy or laparotomy and heightened patient anxiety and occupational absenteeism (6). This case report discusses the presentation of ACNES and aims to make clinicians aware of this differential diagnosis.

## Case presentation

### Patient description

The patient is a 76-year-old male with a significant medical history, including colon cancer, successfully treated with anterior resection in 2005, with no known recurrence. He also has a history of benign prostatic hyperplasia (BPH), treated with tamsulosin, prostate cancer (Gleason score 3 + 4, watchful waiting), and glaucoma, managed with regular ophthalmologic care. The patient is a non-smoker but reports moderate to heavy alcohol consumption, averaging 2–3 glasses of wine daily for several years. He denies recreational drug use, and his dietary habits are described as average, with no known food intolerances or specific dietary restrictions.

At the initial presentation to medical practitioners, the first of which occurred 2 weeks following the onset of symptoms, the patient presented with a main complaint of persistent abdominal pain localized to the right side of the umbilicus, associated with a burning sensation of the skin. The pain increased at night but remained well localized without occasional radiation to other areas. He denied any systemic symptoms such as fever, chills, weight loss, or night sweats. On physical examination, which did not include Carnett's test, the abdomen was soft, with no palpable masses, tenderness, or evidence of guarding. No skin lesions or rashes were observed over the affected area, and vital signs were within normal limits.

### Laboratory tests and findings

To further investigate the patient's symptoms of localized abdominal pain, a comprehensive set of laboratory tests was performed. The results of these were all within normal limits, suggesting no sign of renal impairment or any electrolyte disturbances. Not only was the hepatogram normal, which massively decreased the chances of any liver pathology, but also alkaline phosphatase (ALP) and gamma-glutamyl transferase (GGT) within range showed no laboratory signs of biliary system pathology, whereas normal amylase and lipase results helped rule out any potential pancreatic pathology. The full and differential blood count showed no laboratory signs of anemia, infection, hematologic proliferative disease, or thrombocytopenia. Normal results of C-reactive protein (CRP) ruled out active systemic inflammation or infection. By performing serology tests for *H. pylori* infection and celiac disease (*H. pylori* IgG, tTG IgA, DGP IgG, IgA), it was proven that the two mentioned diseases were not the cause of the patient's abdominal pain.

### Imaging diagnostic tests and findings

#### Abdominal ultrasound

To investigate the cause of abdominal pain, an abdominal ultrasound was indicated. Before the abdominal ultrasound, the work diagnoses were cholelithiasis and cholecystitis. The abdominal ultrasound showed normal liver echogenicity, no focal liver lesion, and normal portal vein flow, which ruled out any liver pathology. There was no biliary duct dilatation, with a normal common duct diameter of 3 mm showing no signs of cholestasis. Cholelithiasis was proven with multiple mobile gallstones seen on ultrasound in the gallbladder. No gallbladder wall thickening ruled out cholecystitis. Both kidneys were normal in size (right 11 cm, left 12.5 cm) with no hydronephrosis, renal calculi, or focal renal lesion. The spleen and the aorta had normal appearances of the visualized portions, and no ascites were found.

#### CT of the abdomen and pelvis

The next diagnostic procedure was a CT of the abdomen and pelvis, which is a good choice for undifferentiated abdominal pain. The technique of the procedure was a post-contrast study. Thoracic bases were clear with no free intraperitoneal gas or fluid. Liver, spleen, pancreas, kidneys, and bowel showed no significant features. The prostate was markedly enlarged and heterogeneous with axial dimensions of 87 mm × 83 mm. CT strengthened cholelithiasis with no evidence of cholecystitis and biliary dilatation. There were no severe bony features and no bony metastatic disease. Thus, after two imaging procedures of ultrasound and CT, there was still no clear cause of the patient's abdominal pain.

#### Colonoscopy

Following the series of inconclusive tests, the patient was referred by the general practitioner to a gastroenterologist who was familiar with the patient's prior history. The specialist recommended a colonoscopy to rule out Crohn's disease as the possible cause of abdominal pain. It is noteworthy that the patient had not been previously diagnosed with Crohn's disease and had undergone regular colonoscopies in accordance with post-colon cancer surveillance guidelines. Apart from the presence of active diverticulitis, the bowel was found to be insignificant and relatively healthy, ruling out Crohn's disease as the cause of recurrent abdominal pain.

## Establishing an ACNES diagnosis

**Figure 1:**
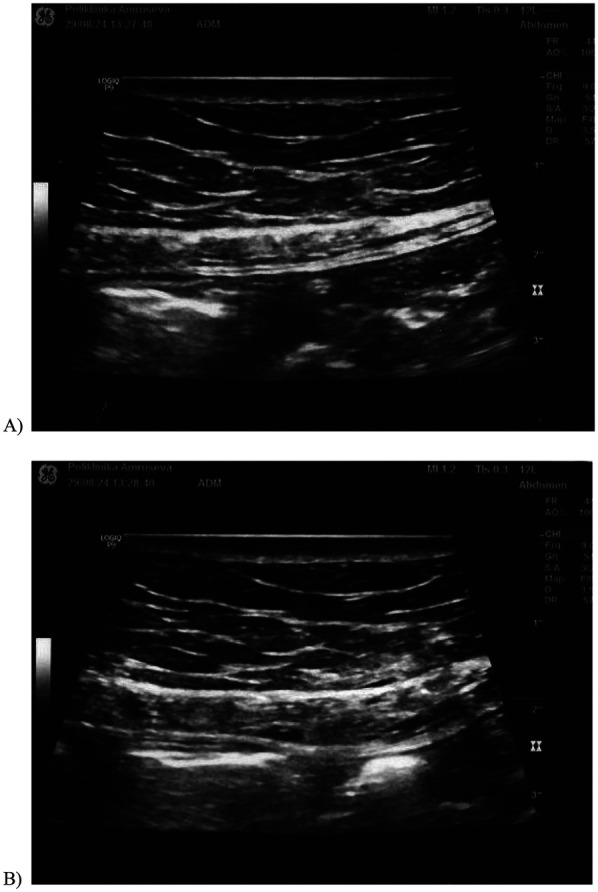
The ultrasound image of the abdominal wall: **(A)** before the block of the anterior cutaneous nerve and **(B)** after the block of the anterior cutaneous nerve.

After the set of laboratory tests, colonoscopy, the abdominal ultrasound, and the CT of the abdomen and pelvis, an anesthesiologist examined the patient. This examination took place nearly 2 months after the onset of abdominal pain. The abdomen status was that the abdomen was soft and without tenderness. After an abdominal palpation, the anesthesiologist performed a test for positive or negative Carnett's sign. The test was performed with the patient in a supine position with the arms crossed over the chest. The patient was then asked to raise his head and feet off the table, while the doctor pushed on the painful spot. If the contraction of the abdominal muscles relieved the pain, the source was probably intra-abdominal, and Carnett's sign would be negative. However, if the source of the pain was in the abdominal wall, the contraction of the abdominal muscles would not reduce the pain and may even worsen it; then Carnett's sign is positive, which was the case in this patient. Additionally, during the examination, the patient localized the pain with his finger. With the positive Carnett's sign and localized pain in the abdominal wall, the anesthesiologist suspected the ACNES syndrome, which was consistent with neuropathic pain presented by the skin burning sensation. These two clinical signs indicated a block of the anterior cutaneous nerve followed by ultrasound control. The patient was placed supine, and the site of maximal tenderness of the abdominal wall was marked. This was followed by skin preparation of the abdominal wall with chlorhexidine and octenisept. A 10 mHz linear ultrasound probe was used (Figure 1A). The medial border of the rectus muscle and the linea alba were visualized, and its lateral end forms the linea semilunaris. The nerve was visualized as a hyperechoic line within the rectus muscle.

Under aseptic conditions, 22 G spinal needle was advanced in the longitudinal axis of the probe. It is mandatory to follow the needle tip all the time to avoid penetration of the peritoneum.

Dexamethasone 4 mg/1 mL + 2% lidocaine 2 mL (total volume of 3 mL) was injected while checking for the spread of the hypoechoic zone in the region (Figure 1B).

The block of the anterior cutaneous nerve confirms the ACNES diagnosis if pain relief occurs after the block. In this case, the patient felt immediate pain relief after the nerve block (the visual analog scale for pain was 2–3/10); therefore, the diagnosis of ACNES was confirmed.

### Management and outcome

After the condition was diagnosed by an anesthesiologist, a structured pain management plan was implemented. Although the localized pain subsided following the anesthetic procedure, the patient continued to experience a severe burning sensation in the skin. To address this, the anesthesiologist recommended initiating treatment with pregabalin. Pregabalin is a CNS medicine that modifies calcium channels to reduce the influx of calcium in the nerves and, therefore, the release of excitatory neurotransmitters. Initial treatment of pregabalin was at a total dose of 50 mg daily, administered as 25 mg in the morning and 25 mg in the evening (1–0–1 scheme) during the first week. The dosage was increased to 75 mg daily in the second week, administered as 50 mg in the morning and 25 mg in the evening (2–0–1 scheme), and 100 mg daily in the fourth week, administered as 50 mg in the morning and 50 mg in the evening (2–0–2 scheme). In addition to pregabalin, a lidocaine patch was applied to the affected area to provide localized pain relief.

After 4 weeks of pregabalin treatment, and with no noticeable effect on the reduction of skin sensation, the patient began a gradual tapering of the drug due to adverse effects mostly related to drowsiness and lack of energy. During the first week of tapering, the total daily dose was reduced to 75 mg. This was followed by a reduction to 50 mg in the second week, 25 mg in the third week, and complete discontinuation in the fourth week.

However, during this period, the patient continued taking neuronal capsules, which are a dietary supplement, at a dose of one capsule daily. Additionally, Neurobion Forte, which is a combination of B1, B6, and B12 vitamins to support nerve repair and regeneration, was temporarily increased to two capsules daily during the first 2 weeks of the tapering process. Both neuronal and Neurobion Forte were prescribed to restore and prevent further nerve damage.

Additionally, due to the recurrence of localized abdominal pain, a second ultrasound-guided nerve block was administered 4 weeks after the initial procedure. This subsequent intervention proved more effective in providing sustained symptom relief.

In addition, the patient also consulted a physiotherapist who provided treatment and guidance on the appropriate exercises that would assist in the process of nerve repair.

As the treatment and exercise progressed, the patient experienced significant pain relief and a normalization of skin sensations. This improvement allowed for the discontinuation of the lidocaine patch, which was replaced by the application of a moisturizing cream to the previously affected region for skin care.

By the conclusion of the treatment, the patient's pain had diminished, although occasional, mild discomfort still recurs in the ACNES area.

## Discussion

The reason behind the entrapment is that the anterior branches of the intercostal nerves enter the rectus abdominis muscle at a perpendicular angle and are, therefore, particularly vulnerable, unlike lateral branches, which enter the muscle at an oblique angle. Any condition that increases pressure on the abdominal wall, as well as the surgical procedures resulting in postoperative scars and adhesions, may damage the tissue and increase the risk of ACNES (3).

In patients diagnosed with ACNES, the administration of systemic drugs is usually the first course of treatment. These include non-steroidal anti-inflammatory drugs (NSAIDs), weak opioids, and antiepileptics (gabapentin) and antidepressants, two drug classes commonly used for neuropathic pain management. The effectiveness of systemic drug therapy alone for treating ACNES is difficult to infer from current studies, given that they are moderately effective (7) in some patients and ineffective in others (8).

Due to the variable efficacy of treating ACNES with systemic medication alone, the majority of cases are eventually treated using interventional techniques, most commonly trigger point injections with 1% lidocaine (9). The nerve block using lidocaine is highly effective, with reports of immediate pain reduction in 83% of suspected ACNES patients (10). Evaluating the efficacy of lidocaine injections for treating ACNES is complicated by the fact that ACNES is diagnosed based on a >50% pain reduction following a 1% lidocaine injection. Therefore, patients who are diagnosed with ACNES are invariably those who respond well to lidocaine. Moreover, recurrence of pain is common in patients treated with local anesthetics only, with up to 25% of diagnosed ACNES patients requiring subsequent injections due to the reappearance of pain (11). The use of lidocaine in ACNES may arguably serve more as a diagnostic rather than a therapeutic tool. Although it cannot always provide full pain relief for patients with ACNES, it does prove that the pain does not originate from a visceral disease but rather the abdominal wall.

In our case, the patient reported no meaningful recurrence of pain at the end of the treatment period. Moreover, it is to our knowledge the first case in which interventional treatment was combined with exogenous factors important for repairing nerve damage, thus aiming to repair the cause of the pain, as opposed to purely managing the symptoms (i.e., abdominal pain).

One of the medications used for this purpose is a combination of vitamins B in the form of Neurobion Forte (vitamin B1, B6, and B12). The main and preferred source of energy for the nervous system, including peripheral nerves that may become pinched to cause ACNES, is carbohydrates. Thiamine (vitamin B1) in the form of thiamine pyrophosphate is essential for feeding the end product of glucose breakdown in glycolysis, pyruvate, into the oxidative energy metabolism to produce adenosine triphosphate (ATP). Moreover, it has been reported that vitamin B1 may further aid nerve metabolism and health by acting as a site-directed antioxidant, protecting from oxidative damage (12). Highlighting a specific role of vitamin B6 (pyridoxine) in maintaining nerve health or enhancing neuronal repair is difficult due to its general, yet critical, role in amino acid metabolism. As such, it is involved in the metabolism of neurotransmitters, specifically GABA and serotonin, and is involved in the metabolism of sphingolipids, essential components of myelin sheaths in peripheral nerves. The nerve-regenerating function of B12 (cobalamin) is well-documented and proven by numerous studies (13). The demand for B12 is significantly increased during nerve regeneration and myelin formation, due to its essential role in the folate-dependent methionine cycle. In the absence of adequate amounts of B12 during this process, myelin basic proteins can fail to be produced, and homocysteine accumulates, promoting oxidative stress and damage (14).

## Conclusion

ACNES may present as a debilitating condition, and its diagnosis is often delayed due to limited awareness and the fact that it falls outside the typical diagnostic scope of specialists commonly consulted for abdominal pain. In patients presenting with abdominal pain, ACNES should be consistently considered as part of the differential diagnosis. As demonstrated in our case, clinicians may often resort to extensive and unnecessary investigations, including laboratory testing, imaging, endoscopy, and even surgical intervention. These approaches not only fail to address the underlying cause but can also contribute to psychological distress and increased healthcare costs. Given that ACNES can often be diagnosed through a simple bedside maneuver such as Carnett's test, it is essential that this condition be systematically ruled out before proceeding with invasive diagnostic or therapeutic measures in cases of undifferentiated abdominal pain.

## Patient perspective

Although approximately 10 weeks elapsed between symptom onset and diagnostic confirmation, this period was particularly challenging for the patient, not only due to physical limitations, but also from a psychological standpoint (Table 1). During this time, differential diagnoses ranged from Crohn's disease and cholelithiasis to spinal pathology. Given the patient's history of colon cancer, he was concerned about the possibility of a more serious underlying condition. This anxiety was compounded by initial consultations with multiple clinicians, none of whom could provide a definitive diagnosis.

**Table 1:** Timeline—from the onset of the symptoms to definitive diagnosis.

Initial signs	Consultations with GP1	Consultations with GP2	Gastroenterologist	Abdominal surgeon	First visit to an anesthesiologist	Second visit to an anesthesiologist
Pain in the right hemiabdomen, more prominent during physical activity, coughing, and laughing	Initial consultation; referral to abdominal ultrasound and laboratory tests; review of results	Initial consultation; referral for CT; review of results and referral to a gastroenterologist	Physical examination, colonoscopy; referral to an abdominal surgeon	Discussion—no diagnosis, but possibility of gallbladder issues and back condition	Physical examination; administration of Lidocaine; pregabalin prescription	Physical examination; repeat administration of Lidocaine; starting pregabalin tapering; neuronal capsules daily intake 1 × 1; Neurobion Forte 2 × 1
					Neuronal capsules daily intake 1 × 1	
June, second half)	10th July	19th July	9th August	12th August	27th August	26th September

In this case, it was only upon incidental advice from a clinician friend that the patient independently sought consultation with an anesthesiologist, perceiving it as a final step before pursuing more invasive interventions.

At present, the patient has fully resumed normal physical activities and reflects on the ACNES episode with mixed emotions, relief that an accurate diagnosis was eventually made, and concern over the potential consequences had it not been.

## Data Availability

The original contributions presented in the study are included in the article/Supplementary Material; further inquiries can be directed to the corresponding author.
